# Comment on IWGDF ulcer prevention guidelines

**DOI:** 10.1002/edm2.169

**Published:** 2020-07-24

**Authors:** David Scott Nickerson, Stephen L. Barrett

**Affiliations:** ^1^ Northeast Wyoming Wound Clinic Sheridan WY USA; ^2^ US Neuropathy Centers, LLC Marietta GA USA

## Abstract

The IWGDF 2019 Updated Guidelines for prevention of foot ulcers in diabetes advise that nerve decompression surgery not be considered. This nerve decompression option has similar scientific supporting evidence to other surgeries which are recommended. The sanction ignores a large body of non‐Level 1 evidence demonstrating various beneficial outcomes of ND including pain relief, DFU prevention, and protection from recurrence and amputation.

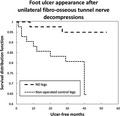

*Diabetes/Metabolism and Related Research* has published the updated IWGDF 2019 *Guidelines on the prevention of foot ulcers in persons with diabetes*. This advisory includes as Recommendation #13, **‘We suggest not to use a nerve decompression procedure, in preference to accepted standards of good quality care, to help prevent a foot ulcer in a person with diabetes who is at moderate or high risk of foot ulceration (IWGDF risk 2‐3) and who is experiencing neuropathic pain’**.^1^ This advice carries a Weak strength of recommendation based upon Low evidence quality. The very brief rationale offered for this guidance points to evidence supporting nerve decompression (ND) being from uncontrolled EBM Level 2 and 3 studies and notes non‐surgical ‘standard of care’ options being available. It is a repetition of similar 2015 guidance but lacks the previous call for more evidence.

We are struck by the inconsistency of this adverse advice with other guidelines, particularly the immediately preceding #11 and #12 recommendations addressing surgical tendon lengthening and flexor tenotomy procedures. Here, the expert panel authors present supportive opinions for tendon surgery despite an identical Weak/Low recommendation, again noting weakness of supporting evidence. Why the differing recommendation despite similar levels of evidence? No data are presented suggesting greater putative risk to ND compared to tendon procedures. And there is no specific call, as in the prior *Guidelines,* to seek further evidence of relative value of ND versus current standard treatments.

So how should this negative recommendation, to *not* use ND, be viewed? Is there evidence that ND carries increased risks of continued pain, repeat ulceration, sepsis, amputation or mortality? Are there published data that show the *accepted standards of good quality car*e show superior outcomes? Sadly, there is neither. While evidence for ND may not be of Level 1 EBM quality, the evidence against its use does not exist above the level of persistent sceptical opinion.^2^, ^3^ Only with stronger science will such contradictory opinions be resolved.

Now consider that the apparent bias against the nerve decompression procedure might be owing to a lack of appreciation of the dual nature of diabetic peripheral neuropathy (DPN) and a differing rationale and target for ND neurolysis in contrast to current standard treatment or the surgical tendon procedures. DPN has long been deemed to have a solely metabolic aetiology labelled length‐dependent axonopathy, and to be both progressive and irreversible. Only recently has come attention to the common presence of secondary, metabolically induced, peripheral nerve compressions in diabetes. This important entrapment component of neuropathy is due to physical nerve trunk enlargement within constrained spaces like fibro‐osseous tunnels and is substantiated by clinical observation, ultrasonography and MRI imaging studies.^4^, ^5^ Fortunately, this secondary compression neuropathy can be addressed successfully. Immediate nerve function improvement accompanies surgical external neurolysis by division of the anatomic fibro‐osseous tunnel structures which constrain nerve trunk pathways.^6^ This is the sufficient justification for ND surgery.

Integration of an anatomic compression understanding into the DFU aetiology and therapy paradigm has encountered persistent opposition.^3^ But good evidence now exists to support this expanded aetiological understanding. This insight also offers a promising opportunity for an innovative assault on diabetes‐associated extremity pain and foot complications. Therapeutically, DPN pain has been treated medically but found significantly resistant to pharmacologic treatments. The interventions for foot complications have focused upon nail and skin care, use of inserts and footwear to minimize pressure concentration and callus, early recognition of skin breakdown, pressure relief for ulcer healing, and stringent adherence to prescribed interventions. In the standard model, the neuropathy is accepted as immutable. Therefore, no value and considerable surgical site infection risk would attend ND. Yet if neuropathy can be ameliorated by ND, this is a revolutionary idea.

We now know that in DPN, local nerve compression and its sequelae need not be permanent nor irremediable. Although ND evidence is mostly Level 2 or 3, clinical, uncontrolled and often retrospective, it is hardly meagre and compares favourably with contrary Level 5 expert opinion. Surgical site infection has been shown to be linked not to a diabetes diagnosis but to the neuropathy.^7^ Nickerson reviewed reports of the use of ND in DPN producing outcome improvements in pain relief, touch and vibratory sensibility, thermal sensation, perineural pressure, electrophysiologic EMG and NCV measures, pedal transcutaneous pO_2_, arterial flow pulsatility, balance, protection from initial DFU, lowest described ulcer recurrence risk and hospitalization for foot infections and amputation.^8^ Since that review, further evidence has been published/addressing delayed mortality, prolonged survival without recurring ulceration, and improved intra‐operative evoked motor potentials.^6^, ^9^ Animal models of induced diabetes with evolving sciatic nerve trunk enlargement and compression demonstrate progressive behavioural, myelination and electrophysiologic changes, but recovery to nearly normal status by 3 months following ND.^10^ This animal modelling closely mimics the course and pathologic findings of human DPN.

Aside from improvements in these many outcome measures, another rarely mentioned phenomenon may be an important DFU correlate which is also responsive to ND. This is the circulation phenomenon called pressure‐induced vasodilatation (PIV). Zwanenburg et al have reviewed the concept and the evidence that local circulation, as measured by cutaneous laser Doppler flowmetry, is normally increased by gradual application of moderate pressure as might exist on pedal skin while standing and walking.^11^ This local vasodilatation effect is on the order of 40% improvement in superficial blood flow. PIV blockade or absence has been demonstrated both in nerve‐injury animal models and clinically in both human spinal cord injury and diabetic neuropathy. It is known that use of innervated pedicled or free flaps to manage human pressure ulcerations has a better survival record, suggesting PIV restoration is useful. In a rat model, PIV is impaired following chronic nerve compression and restored following release. Such recovery of PIV response begs for confirmation in human DPN after ND.

Acute pain is also known to block PIV in animals. The relevance to chronic human DPN pain is unknown. We do know that chronic DPN pain has been responsive to ND in most cases,^12^ so PIV might be involved in this outcome also.

Neurologists have been reluctant to credit the decompression surgery for comfort restoration in DPN, citing potentials for bias or placebo effects and an unexpected bilateral benefit of unilateral ND.^2^, ^3^ Addressing this scepticism, Rozen et al have presented in a 2017 ADA Scientific Session their Level 1 RCT study of ND and DPN pain relief incorporating sham surgery to eliminate bias and placebo risks.^13^ The results show durable VAS pain score reductions from >8/10 to <3/10 which last over 4 years. We anxiously await that study's peer review and publication.

In DFU recurrence, overall expectations from 19 studies are that annual recurrence risk approximates 40%.^14^ Two of the IWGDF Guidelines authors have declared their opinion, absent any examples, that 75% of DFU complications are avoidable. Yet the actual record of high annual recurrence risk belies that optimism. Two published schemes have driven DFU recurrence risk below 10%. Daily plantar temperature monitoring can recognize incipient inflammation preceding skin breakdown and produce recurrences under 10% via activity restriction.^15^ Nerve trunk decompression at fibro‐osseous tunnel entrapment sites was found by 4 authors to achieve DFU recurrence of <5% annually.^8^ Hurdles to validating the surgical ND hypothesis are gathering the Level 1 evidence required to surmount scepticism, amending an incomplete aetiology thesis and treatment paradigm, training enough surgeons to safely treat hundreds of thousands of neuropathic DFU cases annually, and publishing cost‐benefit analysis based on the avoided expense of the next ulcer.

Still, it is an attractive hypothesis, supported by much published literature, to propose that ND effects on pain relief, restoration of protective sensation, and recovery of PIV in combination can explain these patients' observed resistance to initial or recurrent DFU. We may well find that attacking the secondary compression neuropathy will be more successful and cost effective than current measures emphasizing surveillance and pressure management of DPN patients at high risk. But this remains speculation until academia is willing to participate in designing and joining ND research projects. Should ND prove an effective protection from DFU recurrence as proponents have reported, possibilities exist that the minimized DPN, restored protective sensation, and PIV recovery can be engaged to also prevent initial ulcerations, minimize amputations and avoid early mortality.^9^ The millions facing diabetes neuropathy complications would love to hear such prospects for optimism.

Evidence for use of ND in DSP is not yet as scientifically compelling as would be ideal, coming from studies which are not EBM Level 1. Yet it would be our hope that a more open‐minded and informed IWGDF recommendation could be proffered than, ‘We suggest not to use a nerve decompression procedure’. At the very least, no adverse advisory opposing ND surgery should be made absent reports of actual harm or demonstrated superiority of standard care. Otherwise, DPN patients who may be ideal candidates for ND surgical treatment are denied this option to improve their comfort, prevent complications, and extend their life.

## ETHICAL APPROVAL

This article represents the opinions of the authors on a topic of international clinical import and as such does not require any institutional committee or IRB approval.

## DECLARATION

This essay reflects the opinion of the authors based upon personal experience as a diabetic neuropathy patient who has undergone nerve decompression surgery (DSN), a decade of study and review of the pertinent literature (DSN, SLB), and over 10 years of research and experience with ND and its results in treating diabetic neuropathy complications (DSN, SLB).
